# Whole genome sequencing of *Phomopsis asparagi* reveals molecular basis of asparagus stem blight pathogenesis

**DOI:** 10.3389/fmicb.2025.1670056

**Published:** 2025-11-24

**Authors:** Lingtao Duan, Yunpeng Li, Bo Lan, Changfa Yin, Xing Deng, Ziyu Yang, Qinghua Sun, Baojia Li, Yingqing Yang

**Affiliations:** 1 Institute of Plant Protection, Academy of Agricultural Sciences, Jiangxi, China; 2 Jiangxi Provincial Key Laboratory of Agricultural Non-point Source Pollution Control and Waste Comprehensive Utilization, Jiangxi, China; 3 Heilongjiang University, Harbin, China

**Keywords:** asparagus stem blight, *Phomopsis asparagi*, reference genome, high temperature, pathogenic pathway

## Abstract

*Asparagus* (*Asparagus officinalis L.*), a nutritionally and medicinally valuable crop, faces significant yield and quality losses due to stem blight disease caused by the fungal pathogen *Phomopsis asparagi* (syn. *Diaporthe asparagi*). Despite the implementation of various control measures—including agronomic practices, resistant cultivars, chemical treatments, and biological controls—the lack of comprehensive understanding of the pathogen’s molecular pathogenesis has hindered the development of effective management strategies. In this study, we present the first whole-genome assembly of *P. asparagi* (50.94 Mb) through Illumina sequencing, which contains 4,362 predicted protein-coding genes. Functional annotation identified key virulence-associated pathways, particularly those related to oxidative stress response, reactive oxygen species (ROS) metabolism, cell wall remodeling, and programmed cell death (PCD). Given the known temperature sensitivity of disease development, we performed comparative transcriptomic profiling under optimal (25 °C) and heat-stress (32 °C) conditions. Our findings reveal that thermal stress triggers a sophisticated molecular response cascade in *P. asparagi*: initial environmental sensing through WRKY transcription factors and MAPK signaling activates coordinated stress adaptation mechanisms involving ROS generation, DNA damage repair, metabolic reprogramming (lipid and carbohydrate metabolism), proteolytic activity, and cell wall degradation enzymes. This multifaceted response ultimately culminates in host cell dysfunction and PCD, facilitating fungal invasion. This work provides fundamental genomic resources and mechanistic insights into *P. asparagi* pathogenicity, offering new targets for developing science-based disease control approaches in asparagus cultivation.

## 1 Background and overview

*Asparagus* (*Asparagus officinalis L.*), also known as garden asparagus, belongs to the family Liliaceae and the genus *Asparagus*. It is rich in essential nutrients, including dietary fiber, oligosaccharides, amino acid derivatives, vitamins, and minerals (Nishimura et al., 2013; Jaiswal et al., 2014). Additionally, it possesses medicinal properties such as lung-moistening, cough suppression, phlegm elimination, and tumor growth inhibition (Liu, 2001; Chen, 2005; Chen, 2010; Wang et al., 2010; Zhu et al., 2010; Negi et al., 2010). In recent years, with the expansion of asparagus cultivation, the incidence of diseases has increased annually, particularly asparagus stem blight, which severely affects both yield and quality (Yang et al., 2012a; Meng et al., 2013). The causative agent of this disease is the fungus *Phomopsis Asparagi (Sacc.) Bubak* (Meng et al., 2013; Liu et al., 1991), a globally distributed devastating pathogen (Zhang et al., 1995; Yang et al., 2012b; Uecker and Johnson, 1991), often referred to as the “cancer of *Asparagus*.” *Phomopsis* species are hemi-biotrophic fungi. Their hyphae initially attach to plant epidermal tissues and secrete pectinases and cellulases to degrade the plant cell wall. Subsequently, invasive hyphae penetrate host cells, developing fine necrotrophic hyphae that extract nutrients, ultimately leading to cell death and tissue necrosis (Xu et al., 2025). The disease is particularly severe in Asian asparagus-producing countries, such as China, Japan, Thailand, and Indonesia, with China being the most affected. It is prevalent across all major asparagus-growing provinces in China, with higher severity in southern regions compared to the north. Mild infections stunt growth and reduce yield and quality, while severe cases cause premature plant death and complete field devastation (Yang et al., 2012a; Meng et al., 2013; Sun et al., 2023). Provinces such as Guangdong, Guangxi, Fujian, Zhejiang, Jiangxi, Jiangsu, Shanghai, Henan, Shandong, Hebei, and Liaoning report infection rates of 50%–100%, with 10%–30% plant mortality in heavily affected fields, leading to significant economic losses annually (Lu et al., 2024). Current control strategies for asparagus stem blight include cultivation management techniques, disease-resistant breeding, Chemical pesticides, Biocontrol agents (Qu et al., 2021; Yu et al., 2011). However, due to environmental constraints, pathogen resistance, and technical limitations, these methods exhibit incomplete efficacy.

The studies on the functions of ERD2, MoSNF5, ZFC3, and ZFC2 in the pathogenic pathways of *Magnaporthe oryzae* (Peng, 2018; Xu, 2017; Liu, 2016), the genome annotation of *Fusarium graminearum* (King et al., 2017), and the functional research on the sterol transporter BcVast1 and histone demethylase BcJar1 in *Botrytis cinerea* (Ha, 2023; Hou, 2019) have all elucidated the pathogenic mechanisms of the respective fungi, providing a theoretical basis for disease control. Similarly, Banfield’s (2015) study on *Phytophthora infestans* highlighted the importance of investigating pathogenicity pathways. These findings underscore that to better control asparagus stem blight, further research on the pathogenic mechanisms of its causative agent, *P. asparagi*, as well as the disease resistance mechanisms of asparagus, is essential. Prior studies by Lu et al. (2015) and Zhou et al. (2024) focused on the ITS sequences and inter-simple sequence repeat (ISSR) markers of *P. asparagi*, as well as its viral sequences. Additionally, Abdelrahman et al. (2018) conducted a differential gene expression analysis of resistant wild asparagus and susceptible medicinal asparagus infected with *P. asparagi*. However, none of these studies involved a whole-genome analysis of *P. asparagi*. To address this gap, we sequenced and annotated the whole genome and transcriptome of *P. asparagi* to identify pathogenicity-related genes. Studies have shown that in barley, Magnaporthe oryzae Erd2 not only affects asexual development but also influences appressorium penetration and invasive hyphal growth within the host (Peng, 2018). The proteins ZFC3 and ZFC2 regulate mitochondrial gene expression, thereby controlling the growth and development of *M. oryzae* (Liu, 2016). In strawberries, deletion of the *BcVAST1* gene led to increased sterol content and decreased sphingolipid levels in the cell membrane, indicating that the sterol transporter BcVast1 modulates downstream pathogenic mechanisms by altering membrane sterol composition in *B. cinerea* (Ha, 2023). These findings demonstrate the critical role of genomic research in controlling pathogenic fungi.

The occurrence of stem blight requires hot and humid climatic conditions. Since major asparagus-producing regions in Europe and America have cool climates, stem blight is rarely observed in these countries, and related research reports are limited (Sonoda et al., 1997; Davis, 2001; Yang et al., 2014). In asparagus, the initial onset of stem blight occurs from mid-April to mid-May, during which conidia are continuously released from infected plant residues, leading to secondary infections. The peak period of disease incidence spans from early June to early September. As temperatures rise, hyphal invasion and conidial release accelerate, resulting in coalescing lesions or girdling of stems, leading to widespread outbreaks (Sun et al., 2023). We hypothesize that the disease progression is associated with temperature, with more severe infections occurring under high-temperature conditions. Therefore, we conducted transcriptome sequencing of *P. asparagi* infecting asparagus for 5 days under high-temperature (32 °C) and normal-temperature (25 °C) conditions. By analyzing the functional differences in differentially expressed genes, we aim to uncover the molecular mechanisms underlying its pathogenicity under high temperatures, thereby facilitating further research on these related genes.

To address the issue of asparagus stem blight in high-temperature regions, this study employed NovaSeq platform combined with Illumina technology to generate a chromosome-level reference genome for the stem blight pathogen (*P. asparagi*). Comparative transcriptome analysis was conducted on fungal cultures grown at 25 °C and 32 °C to investigate the pathogenic pathways under elevated temperature conditions. The research aims to identify more efficient, environmentally-friendly and cost-effective control strategies, while providing theoretical foundations for enhancing both the nutritional value and economic benefits of asparagus cultivation.

## 2 Methods

### 2.1 Phenotypic observation

The stem blight pathogen strain XJQ-1 (*Phomopsis asparagi*) was isolated and maintained by our laboratory from diseased asparagus plants. The strain was cultured on potato dextrose agar (PDA) medium at 25 °C for preservation. PDA medium was poured into plates at 10 mL per plate. Using a 5-mm cork borer, mycelial plugs were taken from the edge of 5-day-old XJQ-1 colonies and transferred to the center of fresh PDA plates with an inoculation needle. The inoculated plates were incubated at 25 °C and 32 °C for 7 days. After incubation, mycelial plugs were taken from the colony edges and inoculated onto tender asparagus stems. The inoculated stems were maintained at 25 °C and 32 °C for 5 days, with symptom observation conducted subsequently. Each treatment included five replicates.

### 2.2 Sequencing sample preparation

Mycelia were isolated from asparagus stem blight lesions 5 days after inoculation with strain XJQ-1 by cork-boring and sent for sequencing.

### 2.3 Library construction and sequencing

All libraries were sequenced by Allwegene (Beijing, China). Genomic DNA for Illumina sequencing was extracted using the CTAB method. Briefly, DNA samples were fragmented to 350 bp by ultrasonication, followed by end-repairing, A-tailing, and ligation with full-length adapters for Illumina sequencing and subsequent PCR amplification. Finally, PCR products were purified (AMPure XP system), with library size distribution analyzed using an Agilent 2,100 Bioanalyzer and quantified by real-time PCR. Whole-genome sequencing of *P. asparagi* was performed using Illumina NovaSeq PE150.

For transcriptome sequencing, the NEBNext^®^ Ultra™ RNA Library Prep Kit for Illumina^®^ (NEB, USA) was used to construct sequencing libraries with index codes added to each sample. Three technical replicates were performed per library. Library quality was evaluated using an Agilent Bioanalyzer 2,100 system (Agilent Technologies, USA), followed by paired-end 150 bp sequencing on the Illumina NovaSeq 6,000 platform. Raw data were processed using Trimmomatic (v0.39) to remove low-quality bases (average quality score <20 over 4 bp), adapter sequences, and reads shorter than 100 bp (Bolger et al., 2014).

### 2.4 Genome assembly and evaluation

The genome size was estimated using K-mer-based analysis prior to assembly. Clean data obtained after preprocessing were assembled using SOAPdenovo (v2.04) (Li et al., 2008, 2010), SPAdes (Bankevich et al., 2012), and ABySS (Simpson et al., 2009), followed by integration with CISA (Lin and Liao, 2013). The preliminary assembly was refined using gapclose (v1.12) for optimization and gap filling. The final assembly was generated by filtering out fragments shorter than 500 bp, followed by evaluation, statistical analysis, and subsequent gene prediction.

### 2.5 Gene prediction and annotation

Gene prediction was performed using multiple approaches: *de novo* prediction by TransDecoder, Glimmer, and SNAP with PASA based on transcriptome data; Cufflinks prediction using transcriptome data; de novo Augustus (v2.7) prediction (Stanke et al., 2008); and homology-based Genewise (v2.4.1) prediction with reference to related sequences (when available). Results were integrated using EVM and validated through a second round of PASA.

Functional annotation was conducted against general databases including GO, KEGG, KOG, NR, Pfam, and Swiss-Prot. Protein sequences of predicted genes were aligned to these databases using DIAMOND (*e*-value ≤1*e*-5). For each sequence, the top-scoring alignment result (default thresholds: identity ≤40%, coverage ≤40%) was selected for annotation.

### 2.6 Functional enrichment of differentially expressed genes

KEGG and GO functional enrichment analyses were performed for genes differentially expressed under high-temperature conditions compared to normal-temperature conditions.

## 3 Results

### 3.1 Phenotypic analysis

**Figure 1:**
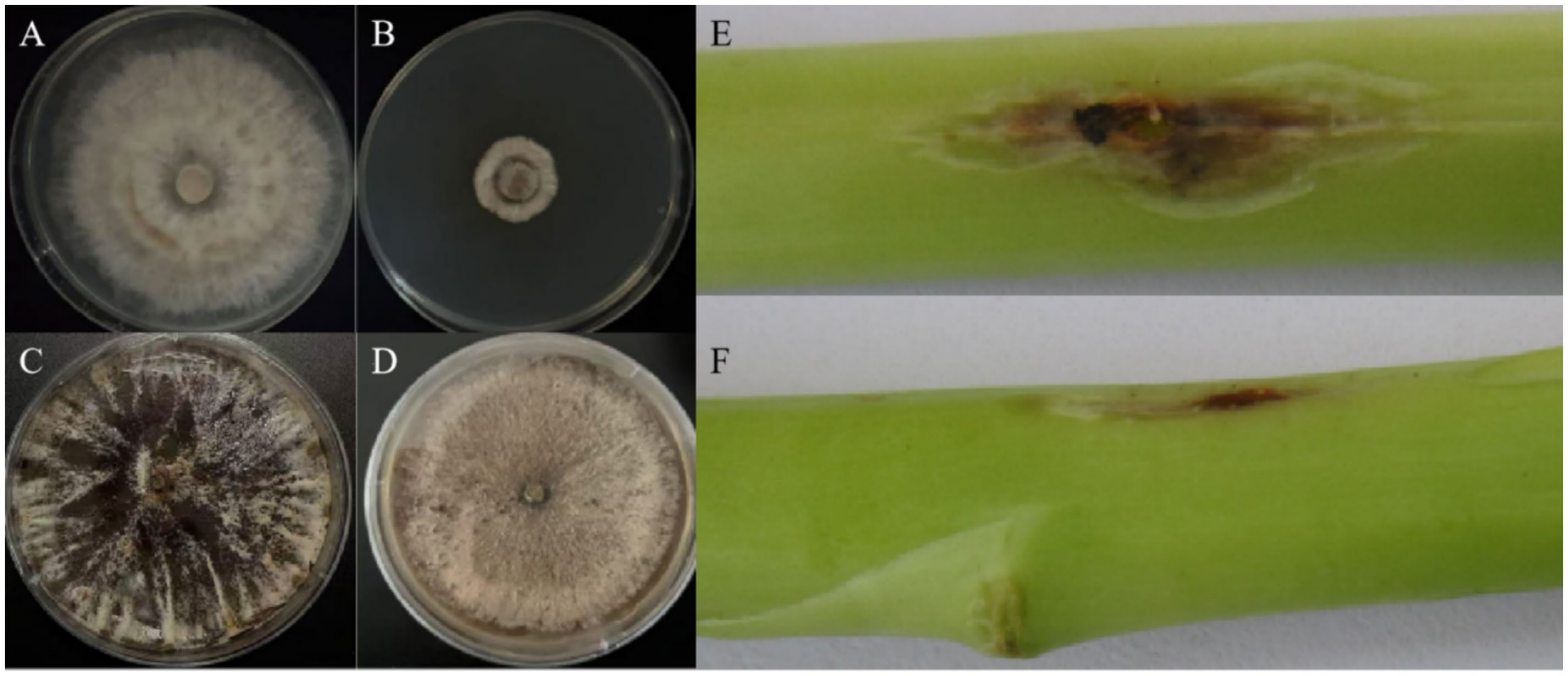
**(A,B)** *P. asparagi* cultured at 25 °C and 32 °C for 5 days, respectively; **(C,D)** *P. asparagi* cultured at 25 °C and 32 °C for 15 days, respectively; **(E,F)** phenotypes after inoculating mycelial blocks of *P. asparagi* cultured at 25 °C and 32 °C for 7 days into *Asparagus officinalis* shoots and then culturing for 5 days.

On PDA plates, the fungal mycelium initially appeared milky white to white, gradually turning grayish-white to light green or dark gray with prolonged incubation (Figures 1A–D). At 25 °C, *P. asparagi* showed rapid initial growth but began to die after 15 days. In contrast, at 32 °C, the fungus grew slowly initially but exhibited accelerated growth after 15 days. Pathogenicity tests using detached asparagus stems showed disease symptoms consistent with field observations (Figures 1E,F). Early infection appeared as small milky white spots that gradually expanded into spindle- or oval-shaped lesions, light to dark brown in color with water-soaked margins. After 5 days of incubation at 25 °C and 32 °C, disease severity was proportional to fungal growth rate.

### 3.2 Genome assembly and annotation

To construct a high-quality reference genome of *Phomopsis asparagi*, we sequenced 5,017 Mb of short reads (Table 1).

**Table 1:** Sequencing data summary.

Sequencing type	Platform	Clean data (Mb)	Insert size (bp)	Read length (bp)
Fungi Survey	Illumina PE150	5,017	350	150
RNA-Seq	Illumina	28.11	350	150

**Figure 2:**
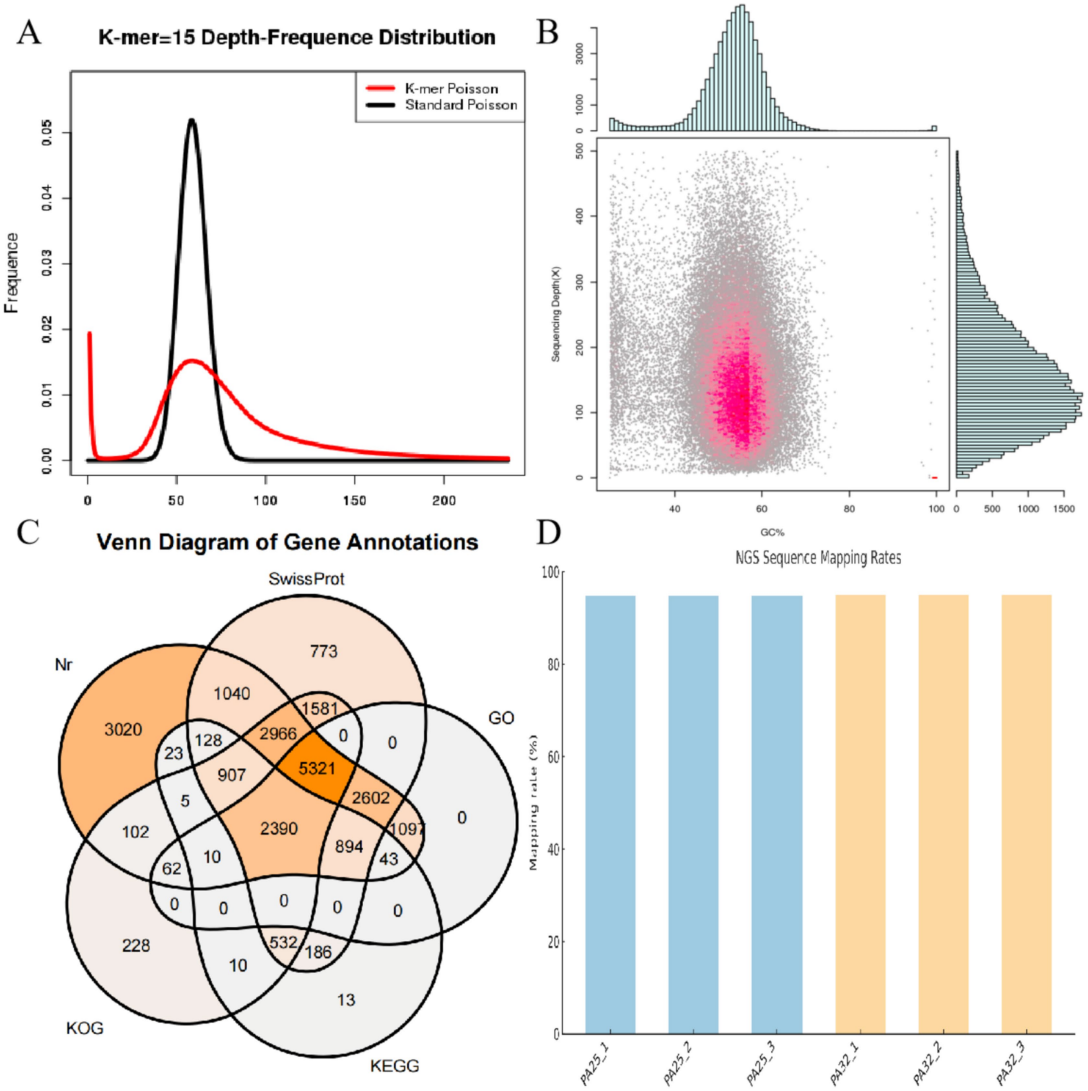
Genomic analysis diagrams. **(A)** 15-mer statistical plot. The abscissa represents k-mer depth, and the ordinate represents the proportion of frequency at each depth to the total frequency. The red curve in the figure is the depth distribution curve of 15-mers from sequencing data, while the black curve is the standard Poisson distribution curve closest to it. **(B)** Statistical chart of correlation analysis between GC content and sequencing depth. The abscissa represents GC content, and the ordinate represents sequencing depth. The right side shows the distribution of sequencing depth, and the upper part shows the distribution of GC content. **(C)** Venn diagram of functional annotations in databases, such as Nr, SwissProt, GO, KEGG, and KOG. **(D)** Alignment result diagram between transcriptome and genome.

Based on 15-mer analysis, the estimated genome size of *P. asparagi* was 58.72 Mb, slightly larger than the assembled genome (Figure 2A). Read alignment to the assembled genome revealed GC bias and abundant repetitive sequences, as indicated by GC content and read coverage depth analysis (Figure 2B). To comprehensively elucidate the pathogenic molecular mechanisms of *P. asparagi*, we achieved high-quality genome assembly and annotation. Gene prediction identified 4,362 protein-coding genes, with functional annotation across multiple databases (Nr, SwissProt, GO, KEGG, and KOG) showing significant overlap (Figure 2C). Notably: 3,020 genes matched Nr annotations, 2,602 genes were assigned GO terms, 894 and 532 entries were annotated in KEGG and KOG, respectively. These results demonstrate high completeness and accuracy of genome annotation. RNA-seq validation further supported transcriptional activity, with >80% of sequencing reads aligning to the genome across samples (Figure 2D), confirming strong consistency between transcriptomic and genomic data.

### 3.3 Prediction of fungal pathogenicity pathways

**Figure 3:**
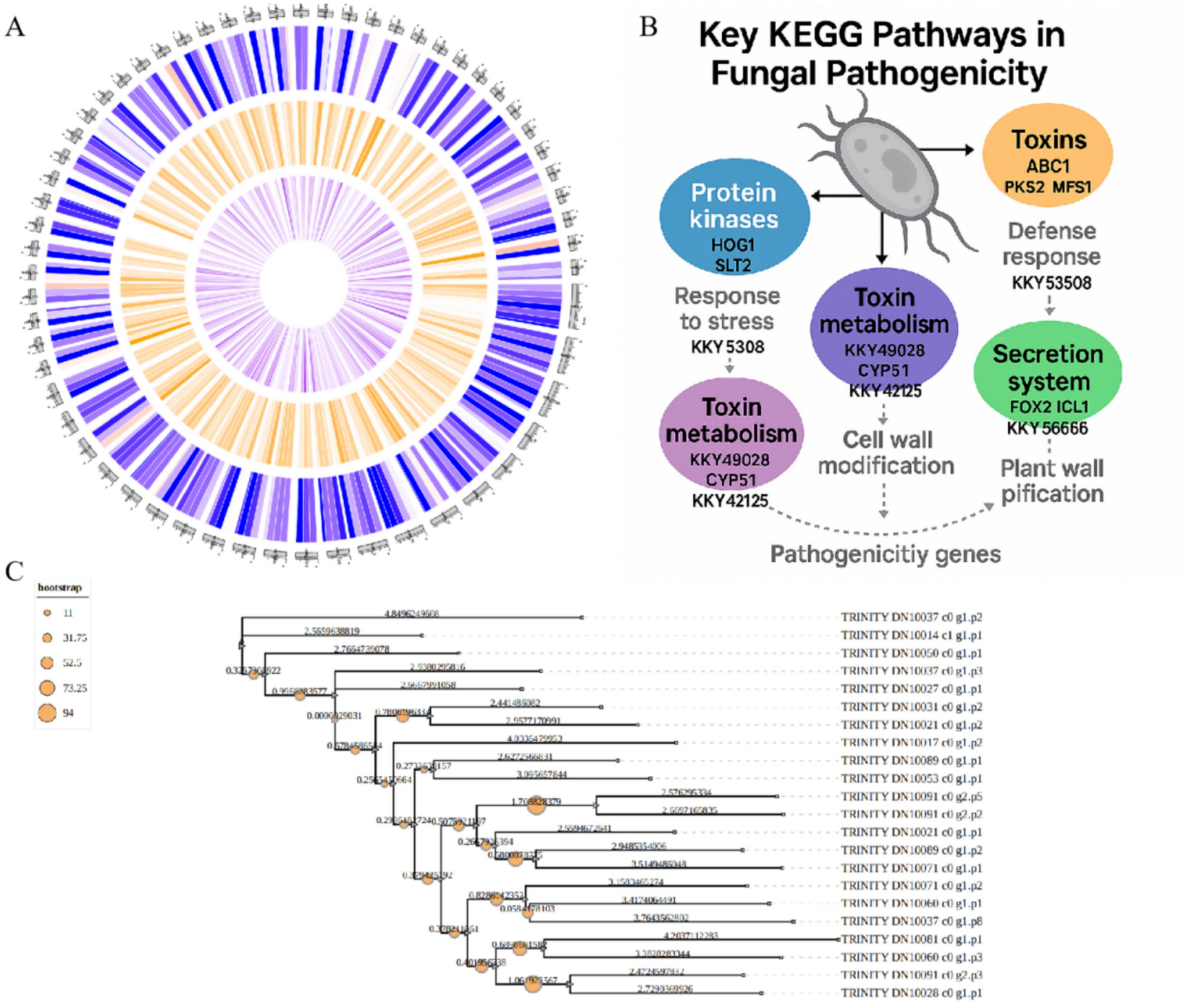
Genomic map and fungal pathogenic pathway diagrams. **(A)** Assembled genomic map of *P. asparagi*. **(B)** Schematic diagram of the fungal pathogenic pathway. **(C)** Phylogenetic tree of *P. asparagi* and its congeneric or closely related plant pathogenic fungi.

The final genome assembly achieved a total length of 50.94 Mb with a GC content of 53.32% (Figure 3A). KEGG analysis of fungal pathogenicity pathways revealed an integrated pathogenic network, encompassing: environmental adaptation (stress response), toxin biosynthesis (toxin metabolism), secretory mechanisms (effector molecule secretion), and host defense disruption (toxin action and cell wall degradation)—collectively establishing fungal pathogenicity (Figure 3B). Phylogenetic analysis demonstrated that *P. asparagi* forms a distinct clade separate from other congeneric or phylogenetically related phytopathogenic fungi (Figure 3C), reflecting its unique genomic composition and potentially specialized pathogenic strategies.

### 3.4 Pathogenic pathways under high-temperature conditions

**Figure 4:**
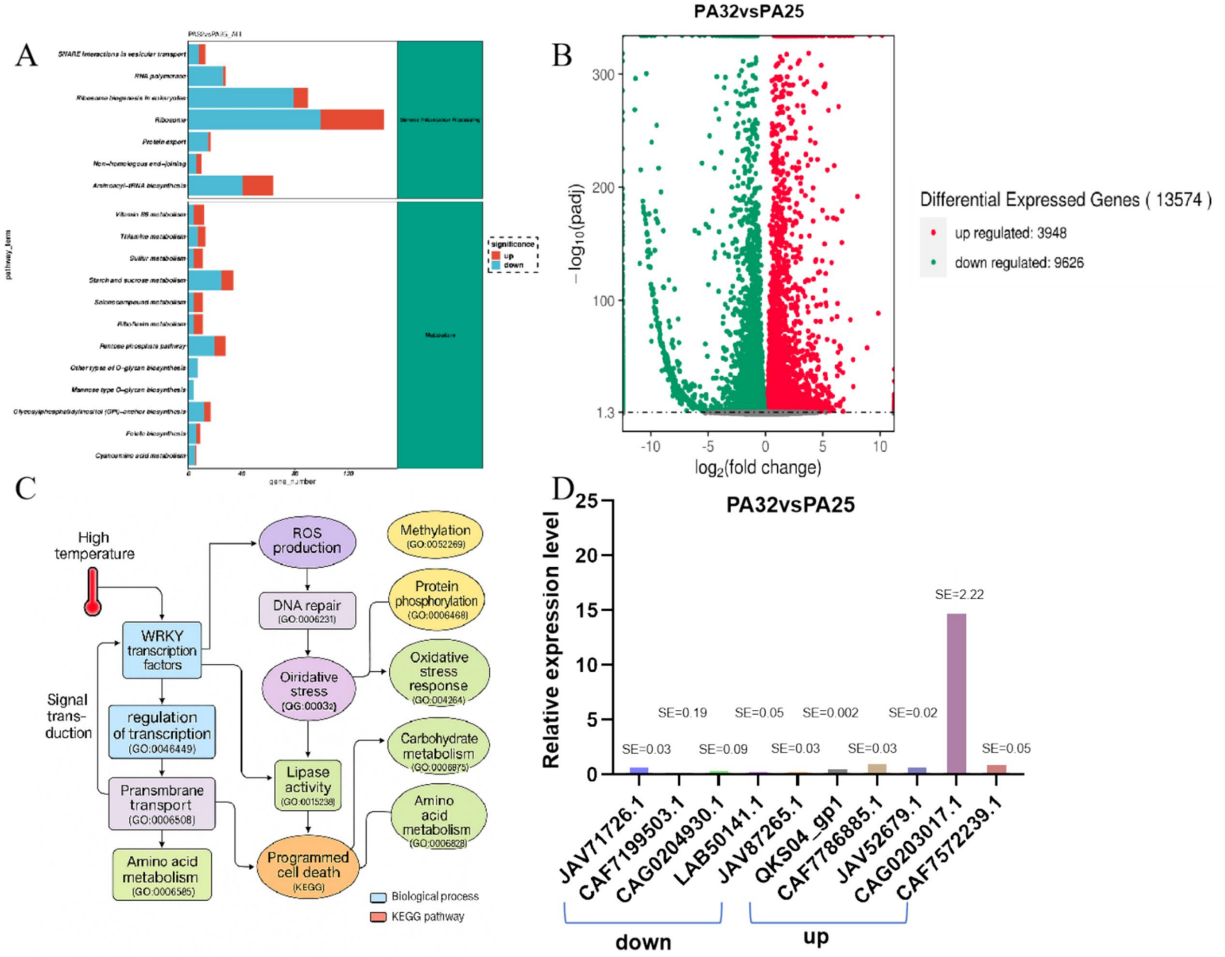
Functional analysis and pathogenic pathway analysis diagrams. **(A)** KEGG functional annotation map. **(B)** Volcano plot of differentially expressed genes in *P. asparagi* cultured at 25 °C and 32 °C. **(C)** Pathogenic pathway diagram under 32 °C conditions. **(D)** Relative gene expression levels under 32 °C and 25 °C conditions.

At the functional level, we simulated host environmental conditions using high-temperature stress as a treatment and identified numerous significantly upregulated differentially expressed genes (DEGs). Volcano plot analysis (Figure 4B) revealed 13,574 significantly differentially expressed genes between high- and normal-temperature conditions, including 3,948 upregulated and 9,626 downregulated genes under high temperature. Relative expression levels of the top five most significantly upregulated and downregulated genes were analyzed (Figure 4D). GO biological process enrichment analysis showed high enrichment of pathogenicity-related pathways, including “oxidative stress response,” “ROS metabolism,” “cell wall modification,” “protein phosphorylation,” “response to fungus,” “signal transduction,” and “programmed cell death” (Supplementary Figures 1, 2). These functional processes are widely involved in virulence regulation, host infection, and stress adaptation in plant pathogens and are considered core modules in pathogenic strategies. KEGG enrichment analysis of all DEGs revealed that in genetic information processing, pathways such as ribosome, ribosome biogenesis in eukaryotes, and aminoacyl-tRNA biosynthesis contained numerous upregulated or downregulated genes. In metabolic pathways, starch and sucrose metabolism and the pentose phosphate pathway showed significant gene expression changes (Figure 4A). Further KEGG pathway analysis enabled construction of a representative pathogenic mechanism map (Supplementary Figure 3), covering pathways such as “MAPK signaling,” “lipase activity,” “proteolytic process,” and “amino acid and secondary metabolite biosynthesis.” Several core genes, including *KKY31689* (encoding a toxin synthase), *KUI66679* (a transmembrane transporter), and *XP_006379301* (an immune regulation-related protein), were identified as highly expressed and enriched in pathogenicity-related pathways, suggesting their potential key roles in regulating virulence expression, secreting active molecules, or disrupting host structures.

To systematically integrate these findings, we combined GO and KEGG pathways to construct a comprehensive molecular network initiated by “high-temperature stress” (Figure 4C). This network reveals that under high-temperature conditions, *P. asparagi* first perceives stress signals through WRKY transcription factors and the MAPK signaling pathway, subsequently activating multiple stress response pathways, including “ROS generation,” “DNA repair,” “lipid metabolism,” “proteolysis,” “carbohydrate metabolism,” and “cell wall degradation.” These ultimately lead to plant cell dysfunction and programmed cell death (PCD). Notably, the network integrates over 20 key GO pathways and highlights representative differentially expressed pathogenicity-related genes, significantly enhancing the interpretability of pathogenic mechanisms through visualization.

## 4 Discussion

Asparagus stem blight is a devastating disease with global distribution, occurring in nearly all major asparagus-producing regions and causing significant economic losses. Previous studies on *P. asparagi* have primarily focused on its biological characteristics, genetic traits, disease epidemiology, and infection processes (Sun et al., 2023; Lu et al., 2024; Qu et al., 2021). Lu et al. (2015) investigated its phylogenetic relationships with similar species through rDNA-ITS sequence analysis, but the molecular mechanisms underlying its pathogenicity remain poorly understood. To address the high incidence and severe damage of stem blight in high-temperature regions, we conducted genomic and transcriptomic studies on the pathogenic fungus *P. asparagi*.

When cultured on PDA medium, *P. asparagi* mycelium initially appeared milky white to white, gradually turning grayish-white to light green or dark gray with prolonged incubation (Figures 1A–D). Pathogenicity assays showed that *P. asparagi* exhibited higher virulence on asparagus under 25 °C during short-term infection, while demonstrating enhanced pathogenicity under 32 °C during prolonged infection (Figures 1E,F).

Genomic analysis revealed a complete *P. asparagi* genome assembly of 50.94 Mb, with 4,362 predicted protein-coding genes. Functional annotation showed 3,020 genes matched Nr database entries and 2,602 genes were assigned GO terms, confirming high completeness and accuracy of the genome annotation (Figures 1A,C,D).

Transcriptome analysis identified differentially expressed genes (DEGs) under 32 °C compared to 25 °C, which were subsequently analyzed for KEGG and GO functional enrichment. GO biological process analysis revealed significant enrichment of pathogenesis-related pathways, including: “oxidative stress response,” “ROS metabolism,” “cell wall modification,” “protein phosphorylation,” “response to fungus,” “signal transduction,” “programmed cell death” (Supplementary Figures 1, 2). KEGG enrichment analysis demonstrated that in genetic information processing pathways, including ribosome, eukaryotic ribosome biogenesis and aminoacyl-tRNA biosynthesis, numerous genes showed differential expression. In metabolic pathways, starch and sucrose metabolism as well as pentose phosphate pathway contained multiple up- or down-regulated genes. The pathogenic mechanism map (Supplementary Figure 3) integrated key pathways such as: “MAPK signaling pathway,” “lipase activity,” “proteolytic process,” and “amino acid and secondary metabolite biosynthesis.” Notably, several core genes *KKY31689* (encoding a toxin synthase), *KUI66679* (a transmembrane transporter) and *XP_006379301* (an immune regulation-related protein) were identified as highly expressed and enriched in pathogenicity-related pathways, suggesting their potential crucial roles in regulating virulence expression, secreting active molecules, or disrupting host structures.

Pathogenicity, as one of the most crucial traits of phytopathogenic fungi, undergoes continuous genetic evolution. The evolution of fungal pathogenicity essentially represents an adaptive process to host plants and their co-evolution (Yang et al., 2020). The virulence of *P. asparagi* is influenced by multiple field conditions including temperature, humidity, asparagus cultivars, and fertilization practices (Sonoda et al., 1997). Our study specifically investigated temperature effects on *P. asparagi* pathogenicity. Under high-temperature stress conditions, *P. asparagi* initially perceives environmental signals through WRKY transcription factors and the MAPK signaling pathway. This subsequently activates multiple stress-response pathways, including: “ROS generation,” “DNA repair,” “lipid metabolism,” “proteolysis,” “carbohydrate metabolism” and “cell wall degradation.” These coordinated responses ultimately lead to plant cell dysfunction and programmed cell death (PCD) (Figure 4C). The findings demonstrate how temperature modulates pathogenic behavior through specific molecular mechanisms in this pathosystem.

Comprehensive analysis revealed that *P. asparagi* possesses a sophisticated pathogenicity regulatory network, where the infection process results from coordinated actions of multiple signaling transduction pathways, metabolic reprogramming, and immune evasion mechanisms. By constructing a systematic mechanistic map, we have for the first time identified the key pathways and genes responsible for virulence induction and cell death under high-temperature conditions, providing a theoretical foundation for future investigations into virulence factor regulation and targeted control strategies. However, this study did not functionally characterize specific genes, and their precise regulatory mechanisms require further investigation.

## Data Availability

The data presented in the study are deposited in the NCBI repository, accession number PRJNA1357034.
