# Supplementary material for: Whole genome sequencing of Phomopsis asparagi reveals molecular basis of asparagus stem blight pathogenesis
Source: Front Microbiol. 2025 Nov 24;16:1670056. doi: 10.3389/fmicb.2025.1670056 (PMC12682896; doi:10.3389/fmicb.2025.1670056)
Supplement: Supplementary file 1 [file Supplementary_file_1.docx]

Supplementary Figures


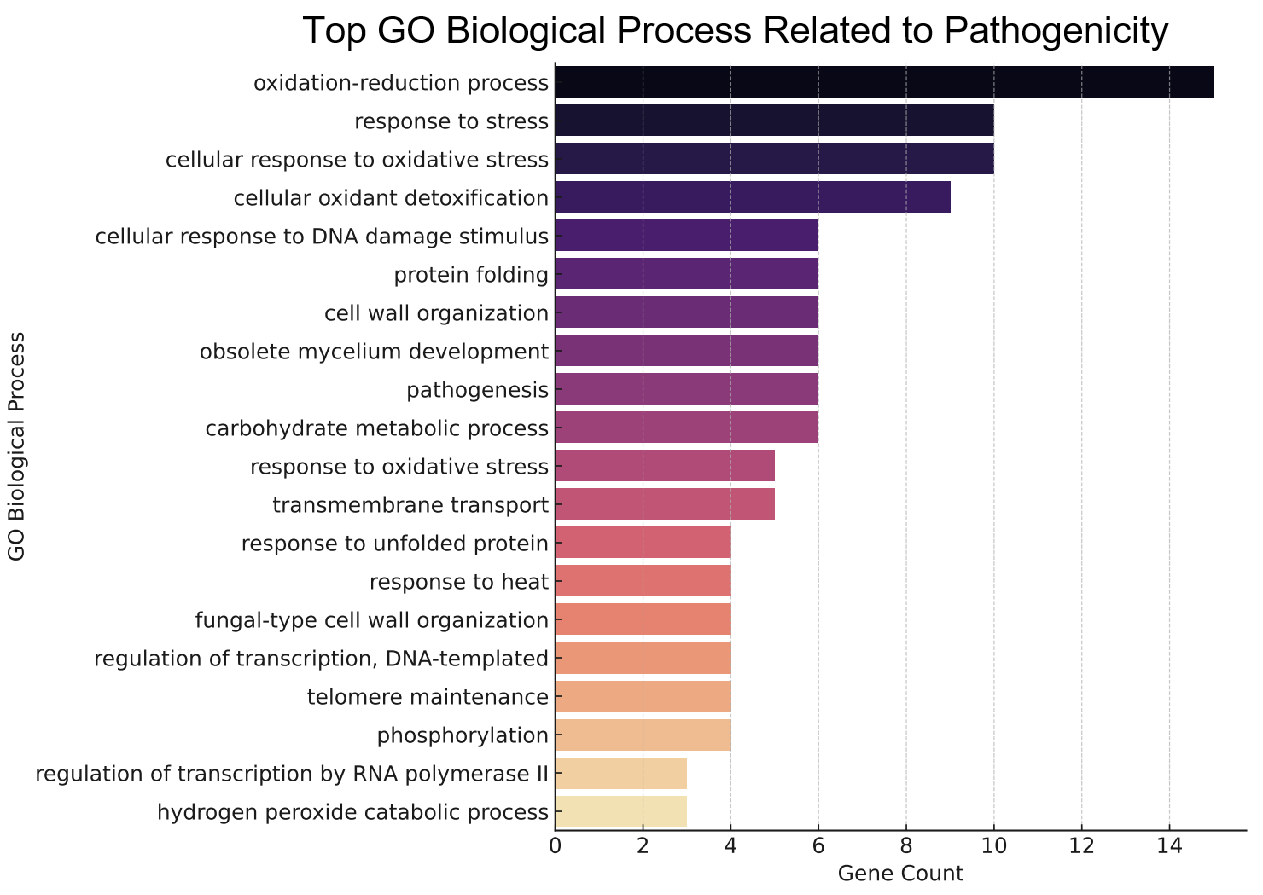


Supplementary Figure 1. GO functional annotation diagram.


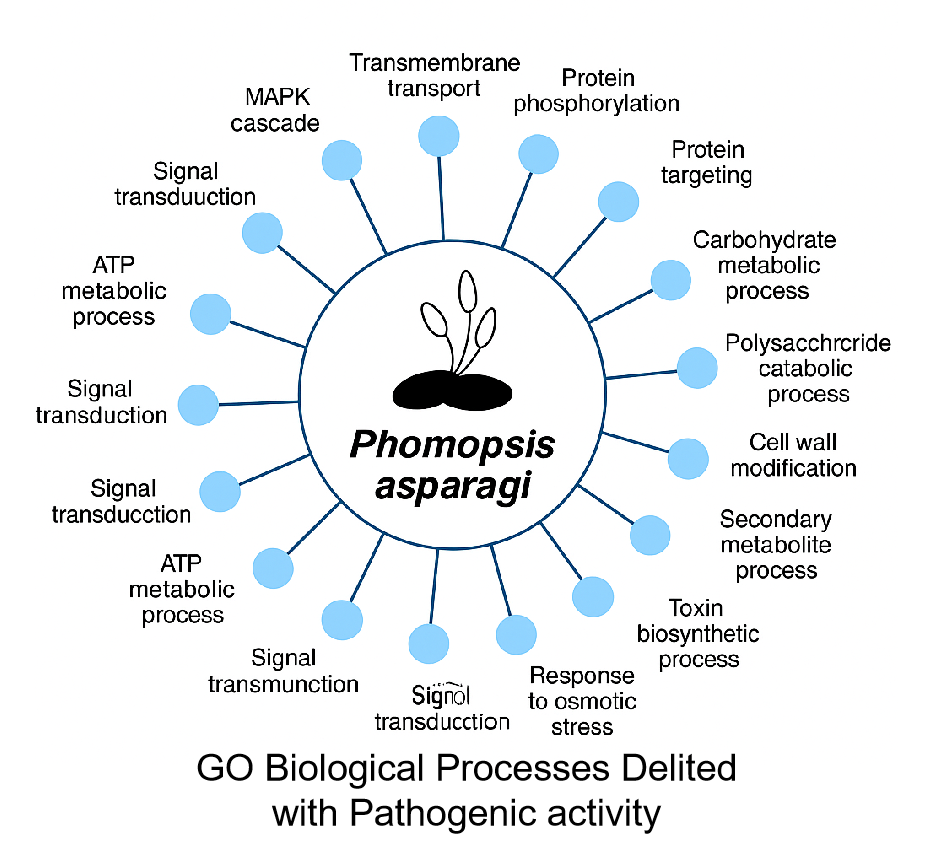


Supplementary Figure 2. GO functional annotation pathway diagram.


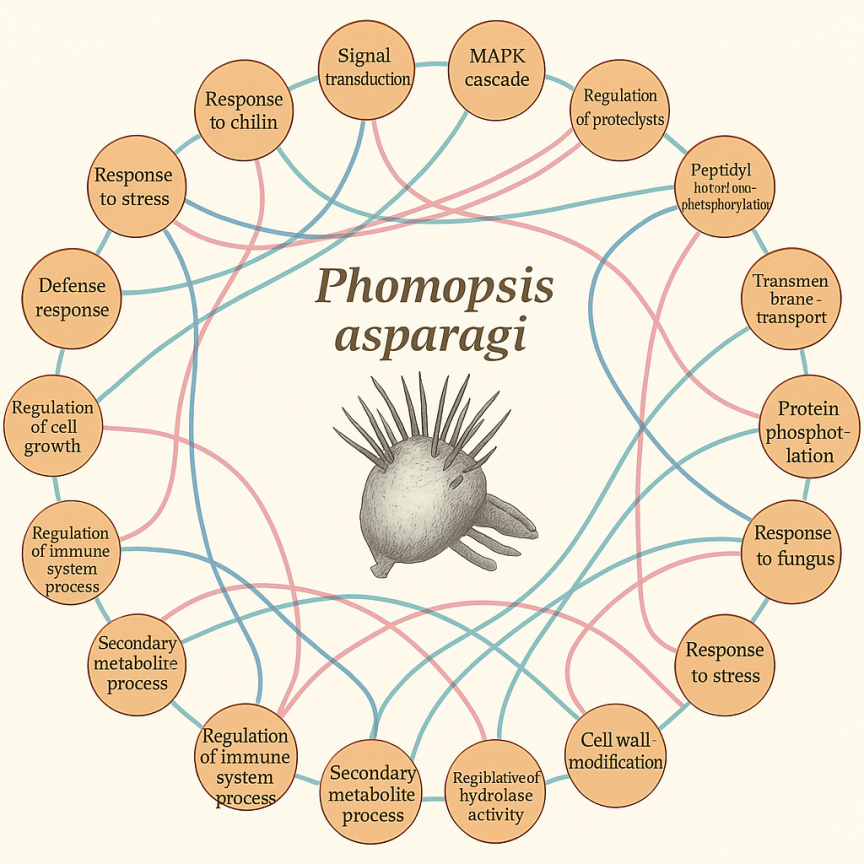


Supplementary Figure 3. KEGG pathway annotation diagram.
